# Endothelin receptor-specific control of endoplasmic reticulum stress and apoptosis in the kidney

**DOI:** 10.1038/srep43152

**Published:** 2017-02-23

**Authors:** Carmen De Miguel, William C. Hamrick, Janet L. Hobbs, David M. Pollock, Pamela K. Carmines, Jennifer S. Pollock

**Affiliations:** 1 Section of Cardio-Renal Physiology and Medicine, Division of Nephrology, Department of Medicine, University of Alabama at Birmingham, Birmingham, AL, USA; 2 Department of Cellular and Integrative Physiology, University of Nebraska Medical Center, Omaha, NE, USA

## Abstract

Endothelin-1 (ET-1) promotes renal damage during cardiovascular disease; yet, the molecular mechanisms involved remain unknown. Endoplasmic reticulum (ER) stress, triggered by unfolded protein accumulation in the ER, contributes to apoptosis and organ injury. These studies aimed to determine whether the ET-1 system promotes renal ER stress development in response to tunicamycin. ET_B_ deficient (ET_B_ def) or transgenic control (TG-con) rats were used in the presence or absence of ET_A_ receptor antagonism. Tunicamycin treatment similarly increased cortical ER stress markers in both rat genotypes; however, only ET_B_ def rats showed a 14–24 fold increase from baseline for medullary GRP78, sXBP-1, and CHOP. Pre-treatment of TG-con rats with the ET_A_ blocker ABT-627 for 1 week prior to tunicamycin injection significantly reduced the ER stress response in cortex and medulla, and also inhibited renal apoptosis. Pre-treatment with ABT-627 failed to decrease renal ER stress and apoptosis in ET_B_ def rats. In conclusion, the ET-1 system is important for the development of tunicamycin-induced renal ER stress and apoptosis. ET_A_ receptor activation induces renal ER stress genes and apoptosis, while functional activation of the ET_B_ receptor has protective effects. These results highlight targeting the ET_A_ receptor as a therapeutic approach against ER stress-induced kidney injury.

Upregulation of the endothelin (ET) system has been reported in a wide range of cardiovascular and renal diseases^1^,^2^; however, the exact cellular and molecular mechanisms through which endothelin-1 (ET-1) leads to renal injury are not fully discerned. ET-1 is an endogenous 21 amino acid peptide with strong vasoactive attributes. The effects of ET-1 are mediated by two G protein-coupled receptors: ET_A_ and ET_B_ receptors. Both receptors bind to ET-1 with the same affinity; however, activation of each receptor subtype leads to opposite physiological and pathophysiological effects. For instance, overactivation of ET_A_ receptors in the kidney promotes renal hypertrophy, fibrosis and inflammation. On the other hand, activation of ET_B_ receptors helps to clear ET-1 from the circulation, as well as stimulating Na^+^and water excretion by inhibition of tubular reabsorption through production of nitric oxide and prostaglandins^3^. Within the renal vasculature, the majority of ET_B_ receptors are located on the endothelium and smooth muscle of the efferent arteriole, whereas ET_A_ receptors are predominant in the afferent arteriole vascular smooth muscle. The distribution of these receptors along the nephron is also distinct: ET_B_ receptors are abundant in cortical and inner medullary tubules, with both ET_A_ and ET_B_ receptors present in tubules of the outer medulla^3^.

Recently, endoplasmic reticulum (ER) stress has been highlighted as a mechanism involved in renal apoptosis and renal injury^4^. ER stress is a type of cellular stress that results from the accumulation of unfolded proteins in the ER. In order to maintain homeostasis, the cell activates the adaptive unfolded protein response (UPR). The ER chaperone protein glucose-regulated protein 78 (GRP78; considered to be the master regulator of the ER stress response)^5^, recognizes the unfolded proteins, physically binds to these proteins, and initializes the 3 parallel arms of the UPR. This leads to the activation of spliced X box-binding protein-1 (sXBP-1), activating transcription factor-4 (ATF-4) and ATF-6, which translocate to the nucleus to temporarily stop further protein transcription and translation; hence, the adaptive UPR gains time for the ER to fold the accumulating misfolded proteins. In case of severe or prolonged ER stress, the cell activates the apoptotic UPR by upregulating expression of the transcription factor CCAAT-enhancer-binding protein homologous protein (CHOP) and caspase-12, leading to cell death *via* apoptosis and eventually inducing organ damage^5^.

Evidence in the literature demonstrates an important role of ER stress in the development of acute kidney injury (AKI) in humans and in animal models of this disease^6^,^7^,^8^. Furthermore, both ET-1 and ER stress are upregulated in renal diseases such as contrast-induced acute kidney injury^9^,^10^, ischemia/reperfusion injury^11^,^12^, septic shock-induced acute kidney injury^13^,^14^, and diabetic nephropathy^15^,^16^, suggesting that overactivation of the ET-1 system may lead to induction of the renal ER stress response. Consistent with this possibility, induction of the UPR by ET-1 has been shown in pulmonary aortic smooth muscle cells^17^ and placental tissue^18^. On the other hand, other authors suggest that activation of the ER stress response mediates ET-1 release from aortic endothelial cells during endothelial dysfunction^19^.

It has been reported that renal injury is preceded by tubular apoptosis and loss of nephrons^20^, and several vasoactive peptides have been implicated in the regulation of cellular apoptosis. However, there are contradictory reports in the literature regarding the role that ET-1 plays in the development of apoptosis and renal injury, with some reports indicating that ET-1 induces cellular apoptosis^21^,^22^ and others suggesting the opposite^23^,^24^,^25^.

The present studies aimed to clarify the role of the ET-1 system in the development of renal ER stress and apoptosis utilizing the ER stress inducer tunicamycin. Similar to other agents mediating kidney damage, such as cisplatin or adriamycin, tunicamycin is commonly used to model antibiotic-mediated acute kidney injury^26^,^27^,^28^. Tunicamycin induces ER stress by inhibiting protein glycosylation and preventing correct protein folding, which results in protein accumulation in the ER and activation of the ER stress response^29^. We hypothesized that the ET-1 system contributes to the development of tunicamycin-induced renal ER stress and apoptosis. Through genetic and pharmacological approaches, we demonstrate that activation of the ET_A_ receptor is important for the induction of apoptosis and the ER stress response in the kidney early in the progression of tunicamycin-induced injury. We also demonstrate the protective role of functioning ET_B_ receptors against tunicamycin-induced renal ER stress and apoptosis.

## Results

### Assessment of the systemic and renal ET-1 system in response to tunicamycin

**Figure 1:**
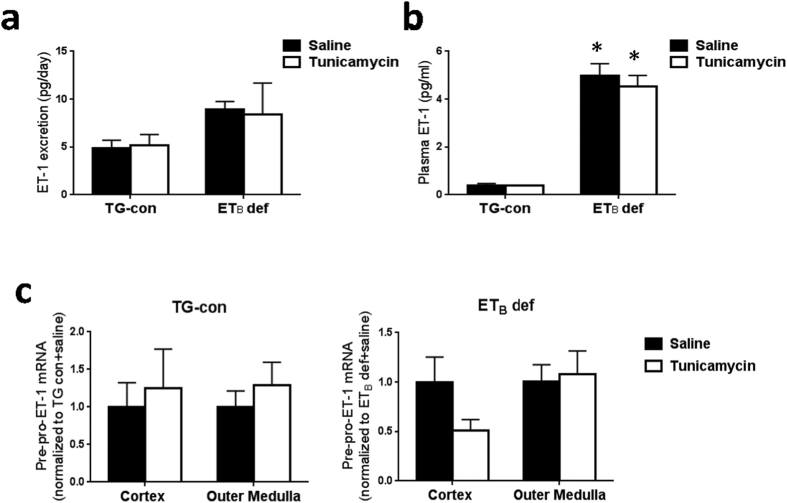
Tunicamycin does not alter the overall systemic ET-1 system in TG-con or ET_B_ def rats. (**a**) Urinary excretion of ET-1 in TG-con and ET_B_ def rats treated with saline or tunicamycin; *n* = 4–5/group. (**b**) Plasma ET-1 levels in TG-con and ET_B_ def rats treated with saline or tunicamycin; *P < 0.05 vs. TG-con + same treatment; *n* = 4–5/group. (**c**) Relative mRNA expression of pre-pro-ET-1 in renal cortex and outer medulla from TG-con and ET_B_ def rats after treatment with saline or tunicamycin; *n* = 4–5/group. RNA expression was normalized to same genotype + saline. Statistical significance was determined by two-way ANOVA with Tukey *post hoc* test.

To study the role of ET-1 receptors in the development of renal ER stress and apoptosis, transgenic control and ET_B_ deficient rats (TG-con and ET_B_ def rats) were treated with a single i.p. injection of tunicamycin (2 μg/g body weight) or saline and studied 24 hours later. The ET_B_ def rats have a natural occurring mutation of the ET_B_ receptor that renders this receptor dysfunctional^30^. As shown in Fig. 1a and b, ET-1 excretion and plasma ET-1 levels were not significantly changed by tunicamycin in either genotype. Moreover, treatment with tunicamycin did not significantly change mRNA expression of pre-pro-ET-1 in renal cortex or outer medulla of either genotype (Fig. 1c). Thus, tunicamycin does not alter circulating or renal ET-1 levels in ET_B_ def or TG-con rats.

### Assessment of tunicamycin-induced ER stress markers in the kidney

**Figure 2:**
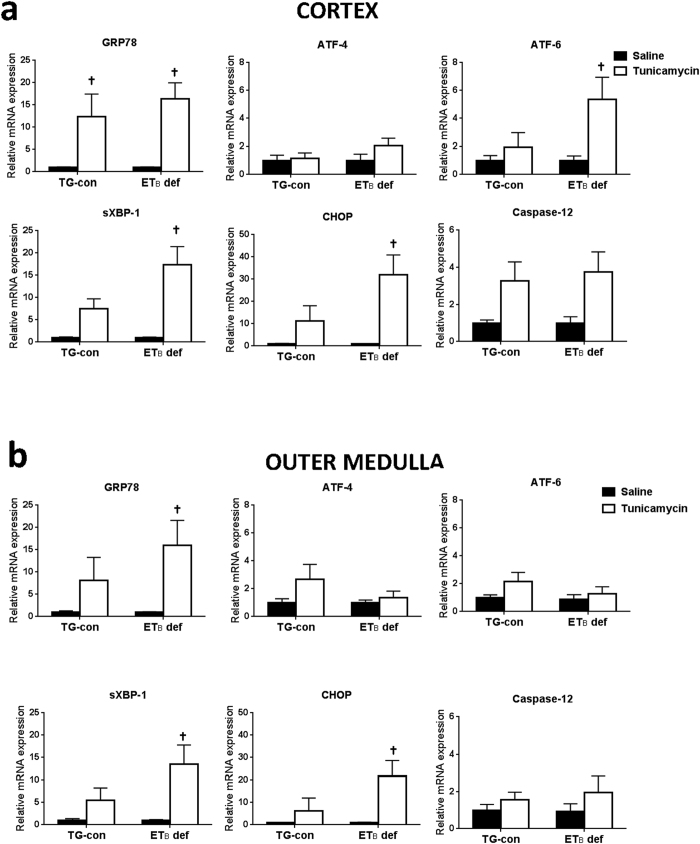
Functional ET_B_ receptors are protective against tunicamycin-induced ER stress development in renal cortex and outer medulla. Relative mRNA expression of ER stress markers in renal cortex (**a**) and outer medulla (**b**) from TG-con and ET_B_ def rats after treatment with saline or tunicamycin. ^†^*P* < 0.05 vs. saline (same genotype); *n* = 6–9/group. RNA expression was normalized to same genotype + saline. Statistical significance was determined by two-way ANOVA with Tukey *post hoc* test.

**Figure 3:**
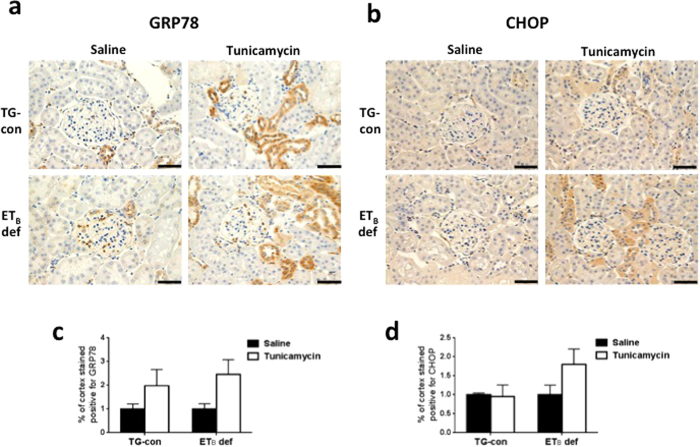
Absence of functional ET_B_ receptors results in prominent GRP78 and CHOP protein expression in renal cortex in response to tunicamycin. Representative images of protein expression of GRP78 (**a**) and CHOP (**b**) in renal cortex of TG-con and ET_B_ def rats treated with saline or tunicamycin. Bar = 50 μm. (**c**) Percentage of cortex stained positive for GRP78 (n = 4–5/group). (**d**) Percentage of cortex stained positive for CHOP (n = 4–5/group).

To explore the potential involvement of ET_B_ receptors in the development of ER stress, mRNA expression of ER stress markers was measured by qRT-PCR in renal cortex and outer medulla of TG-con and ET_B_ def rats treated with saline or tunicamycin (Fig. 2). Preliminary studies showed no changes in expression of ER stress markers in renal inner medulla; therefore, the present studies focused solely on the cortex and outer medulla. TG-con and ET_B_ def rats treated with saline did not differ with regard to mRNA expression of ER stress markers in cortex or outer medulla (Fig. 2). In the renal cortex, TG-con rats responded to the tunicamycin challenge with an 11-fold increase in expression of GRP78 and a 7-fold increase in sXBP-1 expression (*n* = 6–9/group; Fig. 2a). In addition to upregulation of these two markers, tunicamycin treatment of ET_B_ def rats significantly increased expression of three additional markers in this region of the kidney: ATF-6, CHOP, and caspase-12 (with fold increases between 3 and 31; *n* = 6–9/group; *P* < 0.05). Similar to mRNA expression, prominent GRP78 immunostaining was evident in distal nephron segments within the renal cortex of tunicamycin-treated rats of both genotypes (Fig. 3a and c). CHOP immunostaining in the renal cortex of ET_B_ def rats appear most prominent in distal nephron segments and not as prominent in TG-con, although this difference was not significant (Fig. 3b and d).

**Figure 4:**
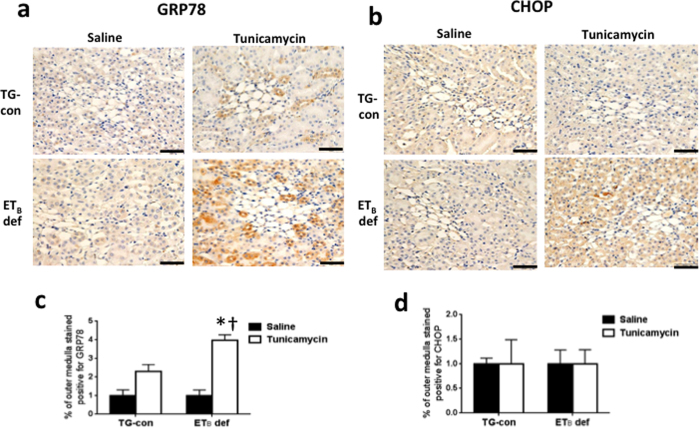
Absence of functional ET_B_ receptors results in prominent GRP78 and CHOP protein expression in renal outer medulla in response to tunicamycin. Representative images of protein expression of GRP78 (**a**) and CHOP (**b**) in renal outer medulla of TG-con and ET_B_ def rats treated with saline or tunicamycin. Bar = 50 μm. (**c**) Percentage of cortex stained positive for GRP78 (n = 4–5/group). (**d**) Percentage of cortex stained positive for CHOP (n = 4–5/group). ^†^*P* < 0.05 vs. same genotype + saline; *P < 0.05 vs. TG-con + tunicamycin. Statistical significance was determined by two-way ANOVA with Tukey *post hoc* test.

The outer medulla exhibited tunicamycin-induced changes in mRNA expression of ER stress proteins only in ET_B_ def rats. These animals responded to the tunicamycin challenge with significant increases in outer medullary mRNA expression of GRP78 (14-fold), sXBP-1 (10-fold) and CHOP (24-fold) (*n* = 6–9/group; *P* < 0.05; Fig. 2b), with no change in expression evident in TG-con rats. The protective effects of the ET_B_ receptor in the outer medulla were also evident at the protein level. As shown in Fig. 4a and c, GRP78 immunostaining was significantly elevated in a subset of outer medullary tubular segments in tunicamycin-treated ET_B_ def rats. Outer medullary CHOP immunostaining tended to be increased with tunicamycin treatment in this genotype (Fig. 4b), however, it was not statistically different from the saline-treated group (Fig. 4d). Tunicamycin did not markedly influence outer medullary GRP78 or CHOP immunostaining in TG-con rats. These results highlight the possible protective effect of the ET_B_ receptor against ER stress in tubular segments located in the outer medulla, as absence of this receptor led to development of tunicamycin-induced ER stress in this area of the kidney.

**Figure 5:**
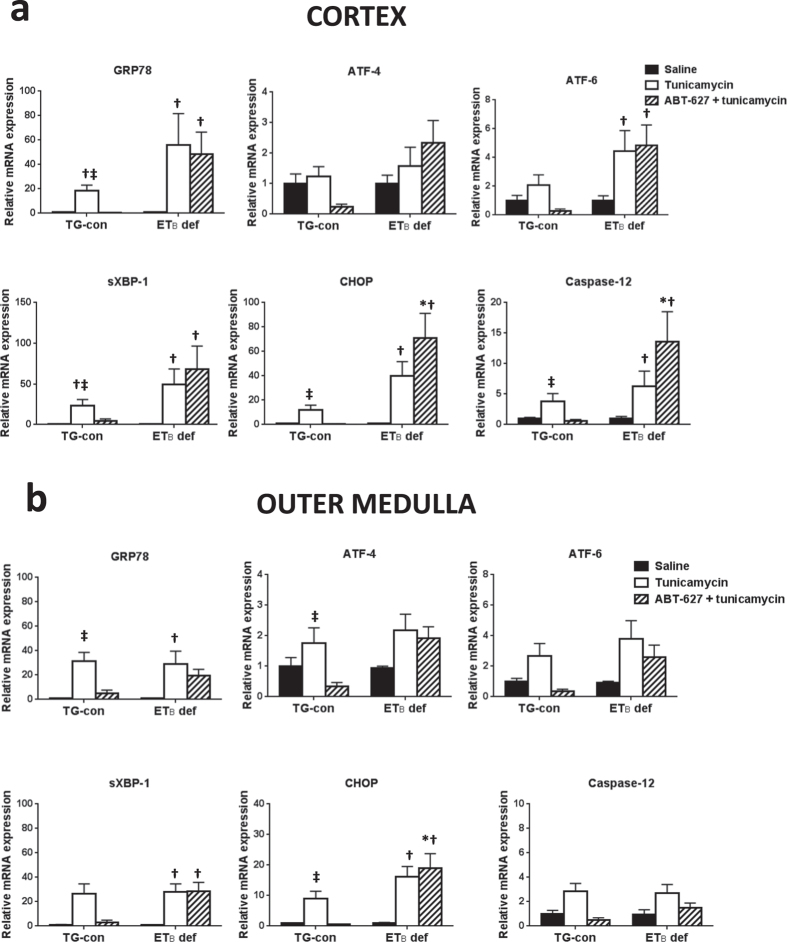
Activation of ET_A_ receptors is important for the development of tunicamycin-induced ER stress in the kidney. Effects of ET_A_ receptor antagonist (ABT-627) on mRNA expression of ER stress markers in renal cortex (**a**) and outer medulla (**b**) in tunicamycin-treated TG-con and ET_B_ def rats. ^†^*P* < 0.05 vs. same genotype + saline; ^‡^*P* < 0.05 vs. same genotype + ABT-627 + tunicamycin; *P < 0.05 vs. TG-con + ABT-627 + tunicamycin. *n* = 6–10/group. RNA expression was normalized to same genotype + saline. Statistical significance was determined by two-way ANOVA with Tukey *post hoc* test.

To assess the role of the ET_A_ receptor in the development of renal ER stress, TG-con and ET_B_ def rats were pre-treated with the specific ET_A_ antagonist ABT-627 (5 mg/kg/day via the drinking water) or left untreated (vehicle), for one week prior to tunicamycin administration. Pre-treatment with ABT-627 significantly blunted cortical and outer medullary expression of GRP78 and CHOP in TG-con rats (GRP78 decreased by 69% in cortex and 78% in outer medulla; CHOP decreased by 77% in cortex and 86% in outer medulla; *n* = 6–10/group; *P* < 0.05). In addition, pre-treatment with ABT-627 significantly blunted expression of sXBP-1 and caspase-12 in cortex (decreased by 77% and 82%, respectively; *n* = 6–10/group; *P* < 0.05), and ATF-4 in outer medulla of TG-con (decreased by 75%; *n* = 6–10/group; *P* < 0.05). Similar trends were apparent regarding ATF-6, although not reaching statistical significance (Fig. 5). These results indicate that activation of the ET_A_ receptor is important for the development of tunicamycin-induced ER stress in the kidney.

In contrast to TG-con rats, pre-treatment with ABT-627 did not protect ET_B_ def rats from tunicamycin-induced renal ER stress, as mRNA expression of ER stress markers remained elevated in both the cortex and outer medulla. Expression of caspase-12 was also significantly elevated in these animals in response to the tunicamycin challenge. These results further support the protective role of the ET_B_ receptor against the development of renal ER stress in response to tunicamycin, as the absence of functional ET_B_ receptors leads to increased expression of ER stress markers in both areas of the kidney regardless of ET_A_ receptor status.

### Assessment of tunicamycin-induced renal apoptosis

**Figure 6:**
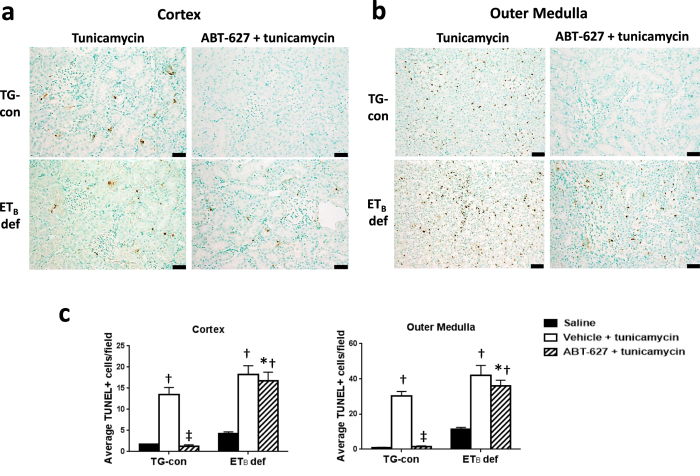
Activation of ET_B_ receptors is protective against the development of tunicamycin-induced renal apoptosis. Effects of tunicamycin on renal apoptosis in renal cortex (**a**) and outer medulla (**b**) of TG-con and ET_B_ def rats pre-treated with vehicle or ABT-627 (apoptosis detected by TUNEL assay). Bar = 50 μm. (**c**) Quantification of TUNEL positive cells in renal cortex and outer medulla. ^†^*P* < 0.05 vs. same genotype + saline; ^‡^*P* < 0.05 vs. same genotype + ABT-627 + tunicamycin; *P < 0.05 vs. TG-con + ABT-627 + tunicamycin. *n* = 5–6/group. Statistical significance was determined by two-way ANOVA with Tukey *post hoc* test.

**Figure 7:**
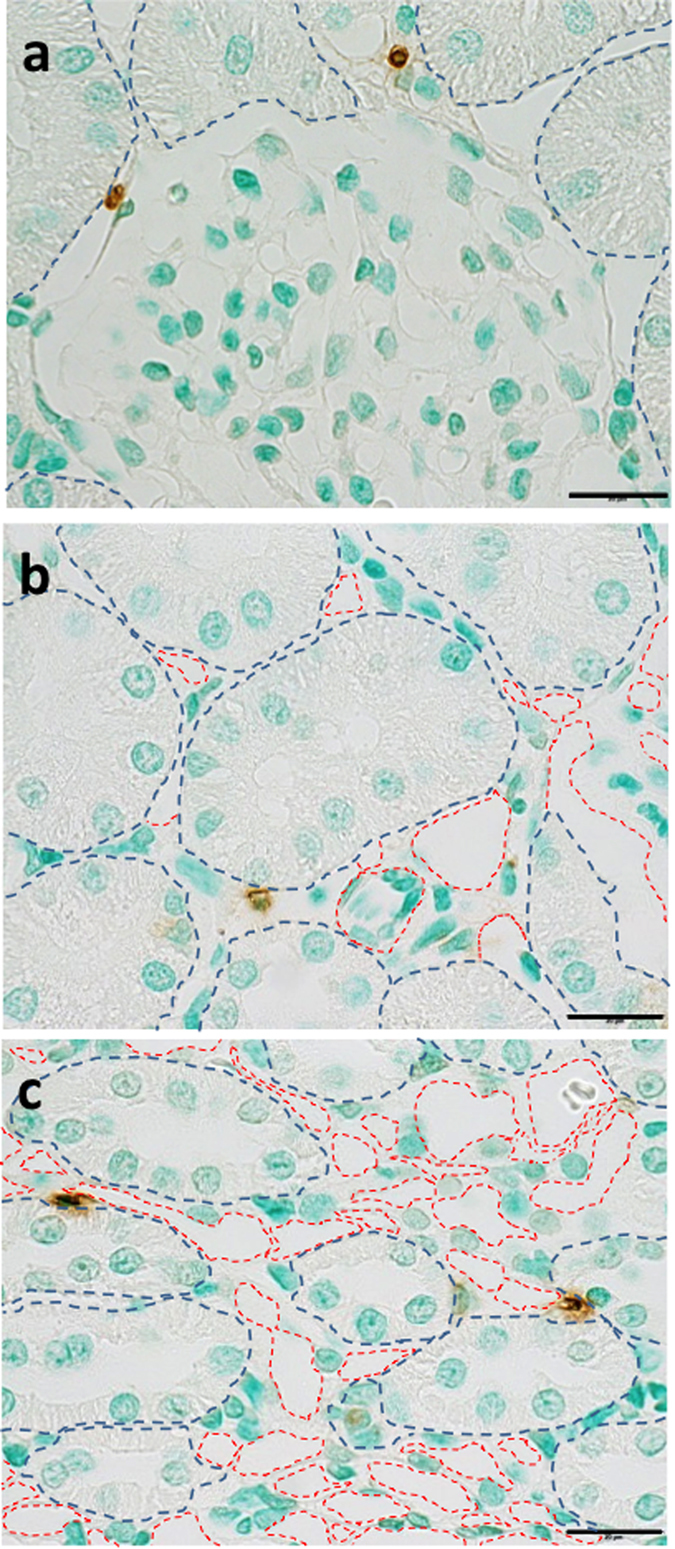
Tunicamycin-induced apoptosis affects interstitial cells located between renal tubules and/or near vasa recta. Representative high magnification images of interstitial apoptotic cells in cortex and outer medulla of an ET_B_ def rat treated with ABT-627 + tunicamycin. (**a**) Glomerular area, (**b**) cortical tubular area, and (**c**) outer medullary vasa recta area. Blue dashed lines outline tubules and red dashed lines outline vessels. Bar = 20 μm.

To assess the role of the ET_A_ receptors in the development of tunicamycin-induced renal apoptosis, TUNEL assay was performed in kidneys from TG-con and ET_B_ def rats receiving ABT-627 via the drinking water for one week prior to the injection of tunicamycin. As indicated in Fig. 6, TUNEL-positive cells were evident both in the cortex and, to a greater extent, in the outer medulla 24 hours after tunicamycin administration to TG-con and ET_B_ def rats. Pre-treatment of TG-con rats with the ET_A_ receptor antagonist almost completely obliterated the tunicamycin-induced apoptosis evident in the renal cortex (decreasing from 13.5 ± 1.6 to 1.3 ± 0.4 TUNEL-positive cells/field; *n* = 5–6/group; *P* < 0.05; Fig. 6c) and outer medulla (decreasing from 30.2 ± 2.7 to 1.6 ± 0.4 TUNEL-positive cells/field; *n* = 5–6/group; *P* < 0.05; Fig. 6c). In contrast, ABT-627 failed to prevent the development of tunicamycin-induced renal apoptosis in ET_B_ def rats, in both cortex and medulla (17.6 ± 2.0 TUNEL-positive cells/field in cortex and 39.0 ± 4.4 TUNEL-positive cells/field in medulla), further highlighting the important role of the ET_B_ receptor in protecting against the development of renal apoptosis. Closer examination of these images at high magnification (Fig. 7) reveals that the TUNEL-positive cells within the renal tissue are not tubular cells, but interstitial cells located between tubules and/or near renal vasa recta.

### Assessment of renal injury and renal function in response to tunicamycin

**Figure 8:**
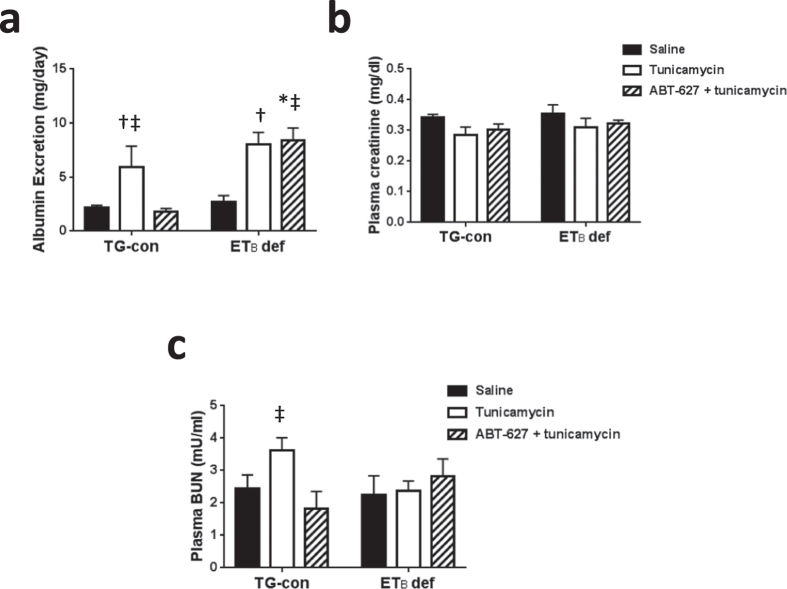
Tunicamycin induces ET_A_ dependent albuminuria in TG-con rats in the absence of changes in renal function. (**a**) Albumin excretion in TG-con and ET_B_ def rats treated with saline, tunicamycin or pre-treated with ABT-627 for one week and then given tunicamycin; *n* = 4–5/group. (**b**) Plasma creatinine levels in TG-con and ET_B_ def rats treated with saline, tunicamycin or pre-treated with ABT-627 for one week and then given tunicamycin; *n* = 6–7/group. (**c**) Plasma BUN levels in TG-con and ET_B_ def rats treated with saline, tunicamycin or pre-treated with ABT-627 for one week and then given tunicamycin; *n* = 3–5/group. ^†^*P* < 0.05 vs. TG-con + tunicamycin. ^†^*P* < 0.05 vs. same genotype + saline; ^‡^*P* < 0.05 vs. same genotype + ABT-627 + tunicamycin; *P < 0.05 vs. TG-con + ABT-627 + tunicamycin. Statistical significance was determined by two-way ANOVA with Tukey *post hoc* test.

To assess whether the acute treatment with tunicamycin increases renal injury, we determined urinary albumin excretion, histological assessments of injury, and renal inflammatory cell numbers. Albumin excretion, a sensitive marker of renal injury, was significantly elevated in both the TG-con and ETB def animals (Fig. 8a). Pre-treatment with ABT-627 prevented tunicamycin-induced increases in albumin excretion in TG-con rats. These effects on albumin excretion were absent in ET_B_ def rats, suggesting that the presence of a functional ET_B_ receptor is important to prevent the development of albuminuria in response to treatment with tunicamycin (Fig. 8a). Immunostaining for ED-1 and CD3 was utilized to assess infiltration of macrophages and T-lymphocytes, respectively, in ET_B_ def and TG-con rats. Numbers of macrophages and T-lymphocytes did not differ between kidneys from ET_B_ def or TG-con rats treated with saline or tunicamycin in any of the studied renal regions (Supplementary Figure 1; *n* = 5/group). Examination of renal histology demonstrated no differences in glomerular sclerosis, interstitial fibrosis or proximal tubule brush border thickness after treatment of both genotypes with tunicamycin (data not shown). However, we observed that tunicamycin led to vasa recta injury in the outer medulla of TG-con and ET_B_ def rats, as indicated by stronger periodic acid Schiff (PAS) staining when compared to the same genotypes treated with saline (Supplementary Figure 2).

In addition, we assessed whether the acute treatment with tunicamycin alters renal function by measuring plasma creatinine, creatinine clearance, and plasma blood urea nitrogen (BUN). Creatinine clearance was unchanged (TG-con vs. ET_B_ def; saline: 2.0 ± 0.3 vs. 2.3 ± 0.2 ml/min, tunicamycin: 2.7 ± 0.2 vs. 2.6 ± 0.5 ml/min), as well as plasma creatinine levels or plasma blood urea nitrogen (BUN) levels in the experimental animals (Fig. 8b and c). Of note, pre-treatment with ABT-627 did not lead to changes in any of these measures of renal function (Fig. 8b and c).

## Discussion

The present study demonstrates that the ET_A_ and ET_B_ receptors play opposite roles in the development of ER stress and apoptosis in the kidney in response to tunicamycin. On one hand, activation of the ET_A_ receptor is important for tunicamycin-induced ER stress and apoptosis in the kidney as well as increased albumin excretion, and, on the other hand, activation of the ET_B_ receptor ameliorates and is necessary for the protection against the renal injury by inhibiting ER stress and renal apoptosis. Despite extensive evidence supporting the role of ET-1 and its receptors in the pathophysiology of kidney disease, the cellular and molecular mechanisms by which this vasoactive peptide mediates the development of renal injury remain unknown. In this paper we demonstrate that the ET-1 system is involved in the development of renal ER stress and apoptosis as well as albuminuria induced by tunicamycin.

The results of the present study indicate that ET_A_ receptor activation is important for the development of tunicamycin-induced ER stress in the kidney. Specifically, pharmacological blockade of ET_A_ receptors with ABT-627 dramatically decreased the expression of ER stress markers in both renal cortex and outer medulla of tunicamycin-treated TG-con rats. Our results agree with previous reports that the ET-1 system is capable of inducing ER stress in cultured pulmonary aortic smooth muscle cells^17^ or placental tissue during pre-eclampsia^18^. Activation of the ET_A_ receptor has been shown to stimulate renal fibrosis, inflammation and increase albumin permeability^31^,^32^,^33^, hallmarks of renal injury which has been linked to ER stress. For instance, it has been reported that inhibition of the UPR response in a well-known model of kidney fibrosis, the unilateral urethral obstruction model, leads to amelioration of fibrosis^34^, and similarly, the three arms of the UPR have been shown to activate the central inflammatory transcription factor, NFκB^35^. At this point we are unsure of how activation of the ET_A_ receptor leads to upregulation of ER stress pathways; however, it has been widely reported that activation of this receptor leads to the production of superoxide by stimulation of the NADPH oxidase^36^. It is also known that oxidative stress can stimulate the UPR as an adaptive mechanism to preserve cell physiology during renal dysfunction^37^. Thus, stimulation of oxidative stress could be a possible mechanism by which activation of the ET_A_ receptor may be leading to the development of renal ER stress. Alternatively, glycosylation of endothelin receptors is important for their function^38^, thus the inhibition of ET_A_ receptor glycosylation by tunicamycin may be affecting the binding of endothelin and/or the specific post-receptor signaling pathways.

Using the ET_B_ deficient (ET_B_ def) rat as an experimental model, the present study revealed the protective role of this receptor against the development of renal ER stress. ET_B_ deficient rats have dysfunctional ET_B_ receptors due to a natural occurring mutation of this gene. Because complete lack of the ET_B_ receptor results in premature death, these rats were rescued years ago by the re-introduction of the ET_B_ receptor in the neuronal tissue; as a consequence, they express functional ET_B_ receptors only in the nerves, while the rest of the tissues (including the kidneys) have non-functional ET_B_ receptors^30^. Because of the importance of the ET_B_ receptor in clearing plasma ET-1, ET_B_ def rats present elevated levels of plasma ET-1 and overactivation of ET_A_ receptors^30^. This phenomenon, in and of itself, is insufficient to provoke ER stress, as expression of ER stress markers did not differ between genotypes in the absence of tunicamycin. However, when presented with a “second hit” of a relatively low dose and a single injection of this ER stress inducer, ET_B_ def rats developed an exaggerated renal ER stress response. This response was especially dramatic in the outer medullary region, where ET_B_ receptors are known to be more abundantly distributed than ET_A_ receptors^39^,^40^. The effects of ABT-627 were absent in the ET_B_ def rats, once again highlighting the protective role of the ET_B_ receptor against the development of renal ER stress. These findings indicate that the ET_B_ receptor opposes the pro-ER stress actions of the ET_A_ receptor and, when the ET_B_ receptor is dysfunctional, the unopposed activation of the ET_A_ receptor leads to an exaggerated ER stress response in the kidney. It is well known that activation of the ET_B_ receptor leads to nitric oxide release^41^,^42^; thus, upregulation of nitric oxide production may be a possible mechanism through which the ET_B_ receptor protects against ER stress development in the kidney.

Tubular apoptosis and loss of nephrons are known to precede kidney injury^20^. Different vasoactive peptides have been implicated in the regulation of apoptosis; however, reports in the literature are contradictory regarding the role of ET-1 in this cellular process. Some studies describe pro-apoptotic effects of ET-1 in vascular smooth muscle cells^21^ or in different parts of the kidney like glomeruli, tubules or interstitial cells^22^. On the other hand, other publications report that ET-1 attenuates apoptosis in fibroblasts^25^, vascular smooth muscle cells^23^ and endothelial cells^24^. The role of ET-1 receptors in apoptosis is also controversial in the literature. Some reports describe pro-apoptotic effects of the ET_A_ receptor in chronic renovascular disease^43^ and polycystic kidney disease^44^,^45^, while others indicate that activation of this receptor promotes cell proliferation and survival during kidney development^46^, in cardiomyocytes^47^ or in vascular smooth muscle cells^48^. Additionally, the loss or inhibition of ET_B_ receptors has been reported as protective against apoptosis in neurons that underwent hypoxia-ischemia^49^, whereas ET_B_ selective agonists led to decreased apoptosis in rat endothelial cells^25^ and in tubules from a mouse model of polycystic kidney disease^45^. Other studies described that the use of an ET_B_ blocker increased apoptosis in rat and human endothelial cells^24^,^50^ and in human melanoma lines^51^.

In addition to effects on the ER stress response, the present study revealed that specific pharmacological blockade of ET_A_ receptor ameliorates tunicamycin-induced renal apoptosis in TG-con rats, while failing to do the same in ET_B_ deficient rats. These results highlight the important role that activation of the ET_A_ receptor has in promoting tunicamycin-induced renal apoptosis. The fact that the renal tubular apoptosis is not diminished by ET_A_ blockade in the ET_B_ deficient rats also emphasizes the protective role of the ET_B_ receptor in opposing the pro-apoptotic effects of the ET_A_ receptor. Interestingly, we found that tubular cells display upregulation of CHOP at the mRNA and protein levels; however, the cells undergoing apoptosis are interstitial cells, rather than the tubular cells. Since tunicamycin treatment did not increase renal infiltration of macrophages or T cells in our acute model, we speculate that the apoptotic cells may be resident immune cells in the peritubular interstitium tissue. Immune cells such as macrophages^52^ or dendritic cells^53^ possess ET_A_ and ET_B_ receptors, are responsive to ET-1, and are also able to synthesize and release ET-1^52^,^53^. Thus, these immune cells may also respond to tunicamycin and activate apoptotic pathways influenced by the ET-1 system. Accelerated macrophage apoptosis induces autoantibody formation and organ damage in lupus nephritis^54^, mainly through increased apoptotic load in the tissue and decreased apoptotic body clearance. Hence, we hypothesize that resident immune cell apoptosis may be the mechanism that leads to the activation of UPR pathways in the renal tubules in our animal model. Further studies are needed to clarify this point.

Although results of the present study indicate that tunicamycin-induced apoptosis is mediated by the ET-1 system, tunicamycin has also been reported to lead to apoptosis through stimulation of oxidative stress^28^, among other pathways. Because ET_B_ receptors counteract the oxidative stress induced by activation of ET_A_ receptors^36^, the renal apoptosis evident in the ET_B_ deficient rats pre-treated with ABT-627 could be due to activation of these alternative pathways by tunicamycin and worsened due to the absence of a functional ET_B_ receptor in these animals.

Finally, these studies also find that this acute tunicamycin treatment induces albuminuria, a sensitive marker of renal injury, in both genotypes but is only ameliorated in the transgenic controls rats with ET_A_ receptor antagonism not in the ET_B_ deficient rats. We documented injury of the vasa recta in both genotypes with the acute tunicamycin treatment. Although other histological measures, such as glomerulosclerosis and tubular fibrosis, were not observed. Further, measures of renal function such as plasma creatinine and BUN, were also not affected by the tunicamycin treatment. These negative findings are most likely due to the acute nature of the experimental protocol.

In conclusion, these findings highlight the potential therapeutic value of specifically targeting the ET_A_ receptor system to prevent the development of antibiotic induced acute renal injury mediated via ER stress and apoptosis. Based on the results presented, we propose that an insult, for instance tunicamycin, stimulates ET_A_ receptors in the tubular epithelium as well as interstitial immune cells, leading to ER stress, apoptosis and, eventually, kidney damage. In this scheme, ET_B_ receptors function as a brake in the system, attenuating the ET_A_ dependent effects on ER stress and apoptosis in the kidney.

## Methods

### Animal studies

All protocols were conducted in accordance with the *Guide for the Care and Use of Laboratory Animals*, and were approved by the University of Alabama at Birmingham and Augusta University Institutional Animal Care and Use Committees. These studies utilized 10–12 week old male ET_B_ deficient rats (specifically, *DBH-ET*_*B*_*;ET*_*B*_^*sl/sl*^ rats) and their *DBH-ET*_*B*_*;ET*_*B*_^+/+^ transgenic littermates. *DBH-ET*_*B*_*;ET*_*B*_^*sl/sl*^ rats (ET_B_ def) express the ET_B_ receptor only in adrenergic tissues, under the transcriptional control of the dopamine β-hydroxylase promoter^30^. In one set of experiments, ET_B_ def rats and their *DBH-ET*_*B*_*;ET*_*B*_^*+/+*^ transgenic littermates (TG-con rats) were placed in metabolic cages for 2 days to acclimate and then received a single i.p. injection of tunicamycin (2 μg/g body weight; Sigma-Aldrich, St. Louis, MO) or saline on the third day. Rats were sacrificed 24 h post-injection, and 24 h urine, plasma and kidneys were collected. In a second set of experiments, 10–12 week old male ET_B_ def and TG-con rats were randomized to receive the ET_A_ receptor antagonist atrasentan (ABT-627; 5 mg/kg/day via drinking water; AbbVie Laboratories, North Chicago, IL) or regular water (vehicle) for 1 week prior to a single injection of tunicamycin (2 μg/g body weight, i.p.). Two days before the injection, the rats were placed in metabolic cages, to allow for urine collection before and after tunicamycin treatment. Twenty four hours post-injection, the rats were sacrificed and plasma and kidneys were harvested; renal cortex and outer medulla were isolated and rapidly snap frozen in liquid nitrogen and kept at −80 °C until further analysis.

### Quantitative RT-PCR

RNA was extracted from renal tissue using RNeasy mini kit (Qiagen, Valencia, CA) and quantified by spectrophotometric analysis (NanoDrop ND-1000, Thermo Scientific, Waltham, MA). RNA was reverse transcribed using Quantitect Reverse Transcription kit (Qiagen) following manufacturer’s instructions. ER stress primers were synthesized by Integrated DNA Technologies (IDT, Coralville, IA; primer sequences are indicated in Supplemental Table 1 55,56,57) and primers for pre-pro-ET-1 were purchased from Qiagen. GAPDH was used as housekeeping gene. RNA expression was detected with Quantitect SYBR green kit (Qiagen) and using a CFX96 Touch RT-PCR detection system (Bio-Rad, Hercules, CA).

### Immunohistochemical analysis

Kidneys were fixed in 4% buffered formalin solution overnight at room temperature, transferred to 70% ethanol for 24 h and paraffin-embedded. Tissues were cut longitudinally into 4 μm-thick sections and mounted on Superfrost slides. Tissue sections were stained with primary antibodies specific for CHOP (1:50; Novus Biologicals, Littleton, CO), GRP78 (1:3,000; Abcam, Cambridge, MA), CD3 (1:400; Abcam), and ED-1 (1:100; Bio-Rad), and detected with polymer conjugated secondary antibody (Biocare Medical, Concord, CA).

Whole kidney scans (100x magnification) were obtained using a scanning microscope fitted with a DP73 camera (Olympus America, Melville, NY), and Metamorph imaging software (Molecular Devices, Sunnyvale, CA) was used to quantify GRP78 and CHOP immunostaining. The cortical and outer medullary areas of each kidney image were outlined using Metamorph software and the amount of positive stain for each antibody was obtained. Data are expressed as the percentage of area of the kidney (cortex or outer medulla) positively stained for GRP78 or CHOP (n = 5/group).

Quantification of renal T-lymphocyte and macrophage infiltration was performed by blindly counting 10 microscopic fields (400 × 400 μm, 200× magnification) in each kidney region (cortex and outer medulla). The numbers are reported as average of the counts in the 10 fields per kidney region.

### Histological analysis

Renal structures were visualized with periodic acid Schiff (PAS), trichrome blue, hematoxylin and eosin, and picrosirius red stains using bright-field microscopy (Olympus BX40; Olympus America). Images were obtained with a digital camera (Olympus DP12; Olympus America). Renal damage was evaluated by assessing glomerulosclerosis, interstitial fibrosis, proximal tubule brush border thickness and vasa recta integrity in a blinded manner. For assessing glomerulosclerosis, ten glomeruli per kidney slide were evaluated and each received a glomerulosclerosis score of 1 = 25%, 2 = 50%, 3 = 75%, or 4 = 100%. Scoring of the degree of thickening of vasa recta was performed by using a presence/absence scale, where a score of 0 indicates no thickening present and 1 means presence of thickened vasa recta. Ten vasa recta bundles per experimental animal were scored, with 5 animals per experimental group analyzed. Data are presented as average of those scores per experimental group.

### Plasma and urinary ET-1

Levels of ET-1 in undiluted samples of plasma and urine were determined by a chemiluminescent assay (Human ET-1 QuantiGlo kit, R&D Systems, Minneapolis, MN).

### Renal function and renal injury marker determination

Plasma and urine creatinine were measured by isotope dilution LC-MS/MS as previously described^58^, and creatinine clearance was calculated. Plasma blood urea nitrogen (BUN) levels and urine albumin were measured by ELISA (Elabscience Biotechnology Co., Bethesda, MD, and GenWay Biotech, Inc, San Diego, CA, respectively).

### Terminal deoxynucleotidyl transferase-mediated dUTP nick-end labeling (TUNEL) assay

Detection of apoptotic cells in renal tissue slides was performed using the Apoptag^®^ Plus Peroxidase *In Situ* Apoptosis Kit (MP Biomedicals, Santa Ana, CA), following manufacturer’s directions. TUNEL-positive cells in tissue sections were counted in 10 microscopic fields (400 × 400 μm, 200X magnification) of renal cortex and outer medulla. TUNEL^+^ counts are reported as average of the counts in the 10 fields per kidney region.

### Statistical analysis

All data are expressed as mean ± SEM. Differences between genotypes and treatments were analyzed by two-way analysis of variance with a Tukey’s *post hoc* test. A *P* value of less than 0.05 was considered statistically significant. All statistical analyses were conducting using GraphPad Prism 6 (GraphPad Software, La Jolla, CA).

## Additional Information

**How to cite this article**: De Miguel, C. *et al*. Endothelin receptor-specific control of endoplasmic reticulum stress and apoptosis in the kidney. *Sci. Rep.* **7**, 43152; doi: 10.1038/srep43152 (2017).

**Publisher's note:** Springer Nature remains neutral with regard to jurisdictional claims in published maps and institutional affiliations.

## Supplementary Material

Supplementary Information
