# Supplementary material for: Endothelin receptor-specific control of endoplasmic reticulum stress and apoptosis in the kidney
Source: Sci Rep. 2017 Feb 23;7:43152. doi: 10.1038/srep43152 (PMC5322462; doi:10.1038/srep43152)
Supplement: Supplementary Information [file srep43152-s1.pdf]

SUPPLEMENTARY INFORMATION FOR "*Endothelin receptor-specific control of endoplasmic reticulum stress and apoptosis in the kidney"* by Carmen De Miguel, William C. Hamrick, Janet L. Hobbs, David M. Pollock, Pamela K. Carmines, and Jennifer S. Pollock.

Supplementary Table 1. Sequence for ER stress primers used for RT-PCR.

Gene name	Sequence
GRP78	F: AAC CCA GAT GAG GCT GTA GCA[56]
	R: ACA TCA AGC AGA ACC AGG TCA C
ATF-4	F: TAT GGA TGG GTT GGT CAG TG[57]
	R: CTC ATC TGG CAT GGT TTC C
ATF-6	F: GAT TTG ATG CCT TGG GAG TC[57]
	R: GGA CCG AGG AGA AGA GAC AG
sXBP-1	F: CTG AGT CCG AAT CAG GTG CAG[58]
	R: GGT CTT GTA GAA GGG TAC CTA
CHOP	F: CCA GCA GAG GTC ACA AGC AC[56]
	R: CGC ACT GAC CAC TCT GTT TC
Caspase 12	F: CAC TGC TGA TAC AGA TGA GG[56]
	R: CCT TCC ATC CGT TCT CAC C

SUPPLEMENTARY FIGURE LEGENDS

Supplementary Figure 1. Treatment with tunicamycin does not alter renal infiltration of macrophages or T-lymphocytes. Average numbers of macrophages (ED-1^+ cells; a) and T lymphocytes ($CD3^+$ cells; b) present per field in renal cortex and outer medulla of TG-con and ETB def rats treated with saline or tunicamycin (n = 5/ group). Statistical significance was tested by two-way ANOVA.

Supplementary Figure 2. Treatment with tunicamycin leads to thickening of the outer medullary vasa recta in both experimental genotypes, as demonstrated by periodic acid Schiff (PAS) staining. Bar = 50 μm. 0 and 1 correspond to absence or presence of thickening, respectively. Statistical significance was tested by two-way ANOVA (n = 5/ group).

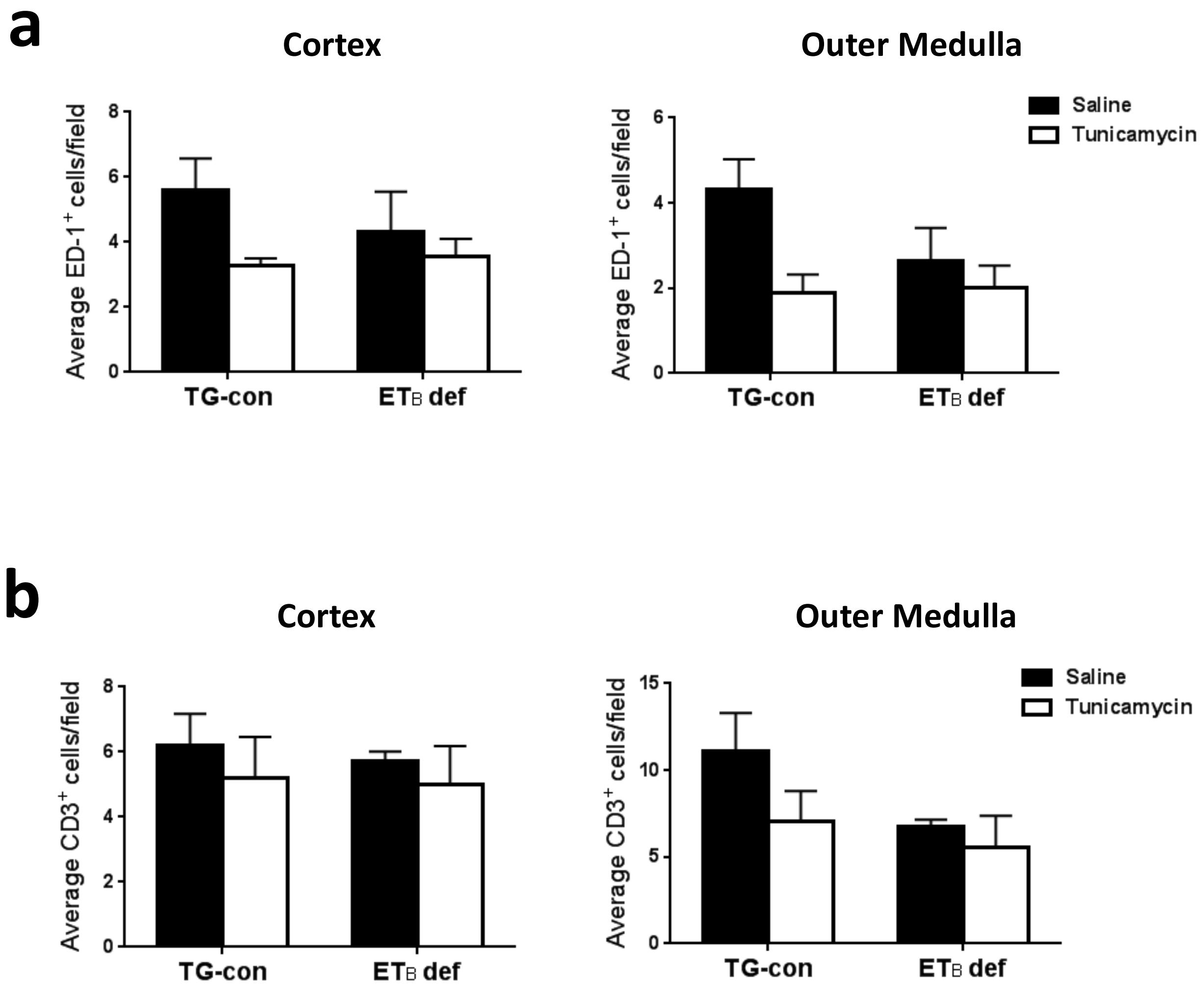
a
Cortex
Outer Medulla
Average ED-1+ cells/field
8
6
4
2
0
6
4
2
0
TG-con
ETB def
Saline
Tunicamycin
b
Cortex
Outer Medulla
Average CD3+ cells/field
8
6
4
2
0
15
10
5
0
TG-con
ETB def
Saline
Tunicamycin

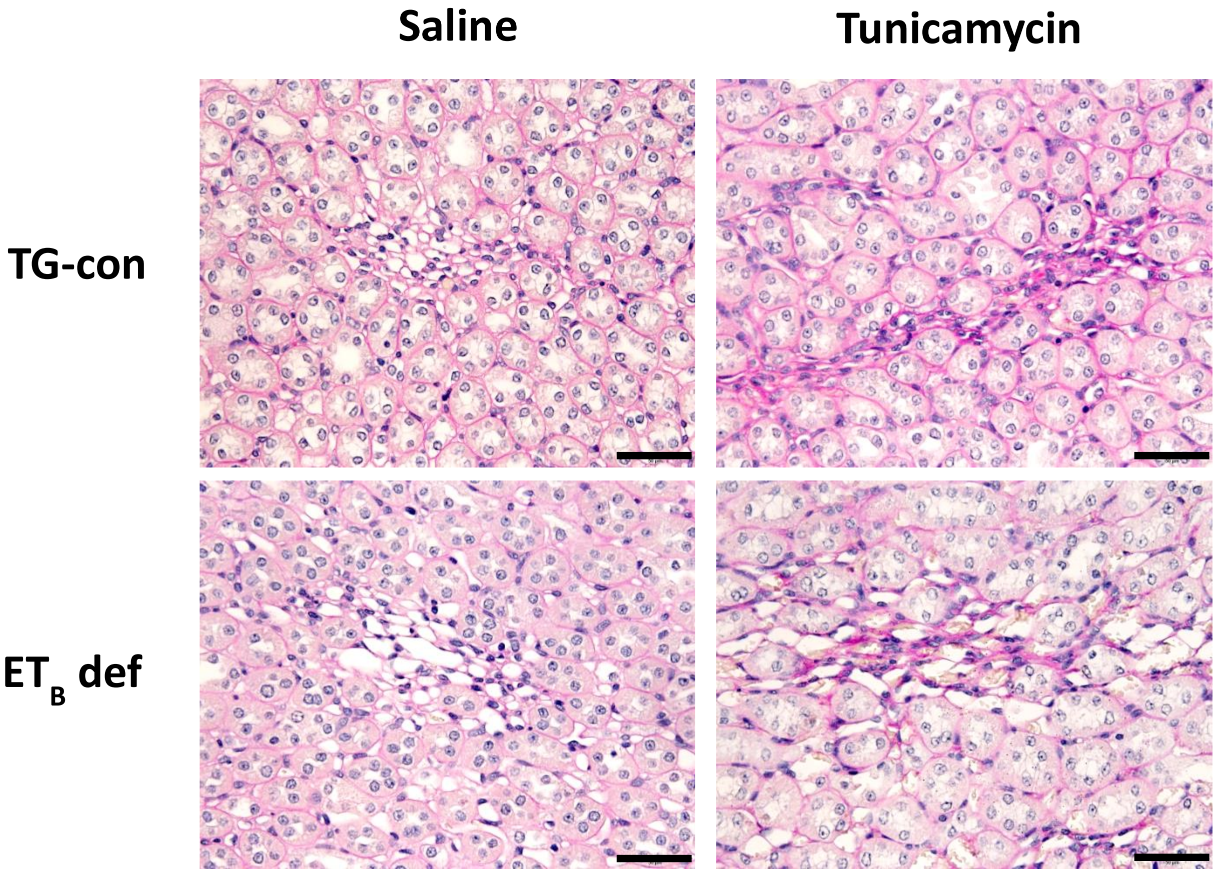
Saline
Tunicamycin
TG-con
ET$_B$ def

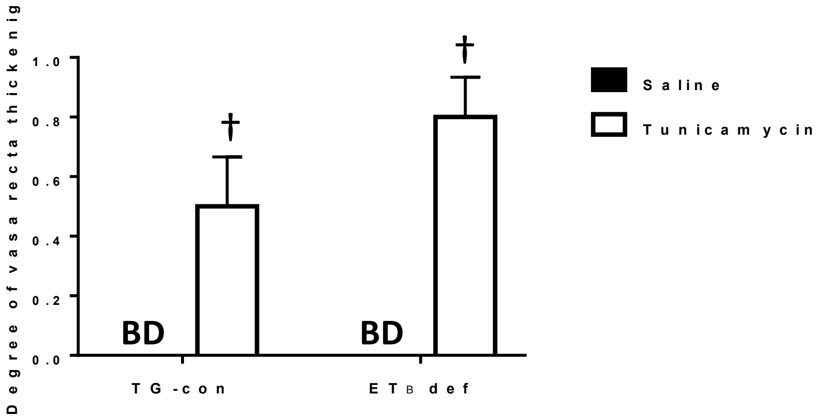
Degree of vasa recta thickenig
1.0
0.8
0.6
0.4
0.2
0.0
BD
BD
†
†
Saline
Tunicamycin
TG-con
ET$_B$ def
